# P99 - Clinical effectiveness of the goat milk-based formula with prebiotics in infants with atopic dermatitis

**DOI:** 10.1186/2045-7022-4-S1-P154

**Published:** 2014-02-28

**Authors:** Svetlana Denisova, Vera Revyakina, Marina Belitskaya, Tatiana Sentsova, Tatiana Malanicheva, Elena Pavlovskaya

**Affiliations:** 1 N.I.Pirogov Russian National Research Medical University, Moscow, Russia; 2 Research Institute of Nutrition, Russian Academy of Medical Sciences, Moscow, Russia; 3 G.N.Speransky Municipal Children’s Clinical Hospital No 9, Moscow, Russia; 4 Kazan State Medical University, Kazan, Russia

## Background

Atopic dermatitis (AD) in most of infants is associated with food allergy; cow milk proteins are a main antigen in 80-90% cases. The aim of study is examination of the clinical effectiveness of the goat milk-based formula with prebiotics in infants with AD aged 6-12 months old.

## Methods

The examination included 50 formula-fed infants with AD aged 6-12 months old. The main group (*n* = 30) comprised infants, who received the goat milk-based formula with prebiotics. The reference group (*n* = 20) – infants, who received a formula based on soy protein isolates. All the patients received standard therapy for AD (antihistamine drugs, local anti-inflammatory therapy, therapeutic and cosmetic skin care, etc.) that did not differ in both groups. The effectiveness of the administered therapy was assessed on the basis of the dynamics of clinical symptoms of disease according to SCORAD score, and also by decrease of total IgE level in serum. The level of total IgE was measured by immune-enzymometric method using the spectrophotometer “Sunrise” (Belgium).

## Results

In the main group, the clinical effectiveness of the formula with prebiotics based on New Zealand goat milk amounted to 76.7% (average duration of the exacerbation period – 13.0 ± 1.5 days, the SCORAD index decreased by 3.7 times – from 30 to 8 scores), in the reference group – 40% (average duration of the exacerbation period – 27.0 ± 1.7 days, the SCORAD index decreased by 2 times – from 34 to 17 scores). The analysis of results of immunologic evaluation revealed that initial level of serum total IgE was increased in 82% patients of the main group, mean level was 260 IU/ml. After therapy the mean level of serum total IgE decreased by 2.7 times – to 95 IU/ml. In the reference group initial level of serum total IgE was increased in 80% patients, after complex therapy it decreased by 1.8 times – from 262 to 145 IU/ml.

## Conclusion

The adapted formula based on goat milk with prebiotics for 6-12 months old infants may be recommended as a dietary product for formula-fed infants with AD.

